# Endocrine therapy and COVID-19 outcomes in women with breast cancer: a nationwide register- based matched cohort study

**DOI:** 10.1186/s12885-026-16152-6

**Published:** 2026-05-12

**Authors:** Aglaia Schiza, Evangelos Digkas, Fotios Papadopoulos, Alkistis Skalkidou, Evangelia Elenis

**Affiliations:** 1 Department of Oncology, Uppsala University Hospital, Uppsala, 751 85 Sweden; 2 Department of Immunology, Genetics & Pathology, Uppsala University, Uppsala, 751 85 Sweden; 3 Department of Medical Sciences/Psychiatry, Uppsala University, Uppsala, 75185 Sweden; 4 Department of Women’s and Children’s health, Uppsala University, Uppsala, 751 85 Sweden; 5 Reproduction Centre, Women’s Clinic, Uppsala University Hospital, Uppsala, 751 85 Sweden

**Keywords:** Breast cancer, COVID-19, SARS-CoV-2, Tamoxifen, Aromatase inhibitors

## Abstract

**Purpose:**

The COVID-19 pandemic posed substantial challenges to cancer care, raising concerns about the safety of ongoing endocrine therapy in women with breast cancer. However, real-world evidence on the association between endocrine therapy and COVID-19 outcomes in population-based cohorts remains limited.

**Methods:**

A nationwide, register-based matched cohort study was conducted in Sweden, including women aged ≥ 55 years who received endocrine therapy for breast cancer during 2020. A total of 31,678 women were included: 8,879 treated with tamoxifen, 21,384 with aromatase inhibitors, and 1,415 with sequential therapy. Exposed women were matched 1:1 by age and region to population controls without breast cancer or estrogen-modulating therapy. Stage-stratified analyses were performed for early-stage and locally advanced breast cancer. Outcomes included COVID-19–related mortality (primary outcome), all-cause mortality, intensive care unit admission, COVID-19–related hospitalization, and laboratory-confirmed SARS-CoV-2 infection.

**Results:**

COVID-19–related mortality did not differ between women receiving endocrine therapy and their matched population controls. Intensive care unit admission risk were comparable across groups. Tamoxifen was associated with lower all-cause mortality in early-stage breast cancer, whereas aromatase inhibitors were linked to higher all-cause mortality and increased COVID-19-related hospitalization in locally advanced disease. A modestly increased risk of SARS-CoV-2 infection was observed among tamoxifen users.

**Conclusion:**

In this nationwide Swedish cohort, adjuvant endocrine therapy was not associated with increased COVID-19–specific mortality. These findings support the continued use of adjuvant endocrine therapy in women with breast cancer without added COVID-19 mortality risk and highlight stage-specific differences in outcomes, reinforcing the safety of endocrine therapy during pandemic conditions.

**Supplementary Information:**

The online version contains supplementary material available at 10.1186/s12885-026-16152-6.

## Highlights

Nationwide Swedish study examining endocrine therapy and COVID-19 outcomes in breast cancer patients.

No increased COVID-19 mortality or intensive care unit admission linked to adjuvant endocrine therapy.

Tamoxifen associated with lower all-cause mortality in early-stage breast cancer.

Findings support safe continuation of adjuvant endocrine therapy during the pandemic.

**Supplementary Information:**

The online version contains supplementary material available at 10.1186/s12885-026-16152-6.

## Introduction

The COVID-19 pandemic severely disrupted global cancer care, causing delays in diagnosis and treatment, particularly in breast cancer [1–4], due to both patient hesitancy and healthcare disruptions, potentially affecting clinical outcomes [5–8].

Patients undergoing active cancer treatment were found to be at higher risk of severe COVID-19 outcomes [9] prompting the introduction of temporarily adapted breast cancer treatment guidelines during 2020. These adaptations included wider use of neoadjuvant endocrine therapy in non-aggressive cases or elderly patients to allow safe delay of surgery [10, 11].

Endocrine therapies may affect immune responses to viral infections, given estrogen’s role in regulating cytokine expression and inflammation through estrogen receptors. Selective estrogen receptor modulators, such as tamoxifen, have been proposed to influence viral entry and immune responses [12–15]. Despite initial concerns that tamoxifen might increase COVID-19 susceptibility, a retrospective study by Bravaccini et al. reported reduced risks of hospitalization and mortality in Northern Italy [16]. However, tamoxifen carries a small but established increased risk of thrombosis, and it remains unclear whether concurrent COVID-19 infection exacerbates this risk [17]. Recent studies suggest that estrogen-containing hormonal treatments may influence outcomes in women with severe COVID-19 infections; however, the evidence remains inconsistent [18, 19]. In parallel, androgen-regulated proteins such as TMPRSS2 have been proposed as prognostic biomarkers for COVID-19 severity [20]. In light of this conflicting evidence, no prespecified hypothesis was formulated.

Given the continued use of adjuvant endocrine therapy in Sweden during the pandemic, a nationwide, population-based study was conducted to evaluate the association between tamoxifen or aromatase inhibitors and COVID-19 outcomes, including mortality, intensive care unit admission, hospitalization, and SARS-CoV-2 infection, with a focus on stage-specific differences.

## Materials and methods

### Study design and data sources

This nationwide, observational, register-based matched cohort study was conducted in Sweden. Data were obtained from prospectively maintained national registers and pseudonymized after cross-linkage using Sweden’s unique 12-digit personal identification number, assigned at birth or upon immigration.

Data were obtained from the following registers:

- National Patient Register: Contains all inpatient and outpatient physician visits and related diagnoses from private and public healthcare providers.
- Prescribed Drug Register: Contains information on Anatomical Therapeutic Chemical codes, drug names, doses, prescription dates, and dispensing details.
- Cause of Death Register: Provides causes, date, and place of death.
- Swedish Cancer Register: Records clinical and morphological cancer diagnoses, tumor spread, and metastases.
- Total Population Register: Contains details on country of birth, sex, marital status, immigration, and emigration.
- Swedish Education Register: Records educational attainment.
- Swedish Intensive Care Register: A national quality register covering intensive care admissions, capturing 100% of severe COVID-19 cases.
- Sminet: A surveillance system for notifiable diseases, recording reports on 60 communicable diseases, including SARS-CoV-2.

Data from the National Patient Register, Prescribed Drug Register, Cause of Death Register, Cancer Register, and Swedish Intensive Care Register were provided by the Swedish National Board of Health and Welfare. The Total Population Register and Education Register were provided by Statistics Sweden, and Sminet data were provided by the Public Health Agency of Sweden.

### Ethical approval and consent to participate

The study was approved by the Swedish Ethical Review Authority (Dnr 2020/03936, date of approval 17 August 2020). The requirement for written or oral informed consent was waived because all data obtained from the Swedish registers were pseudonymized. The public was not involved in this study.

### Study population

The exposed population included all registered females in Sweden who received a prescription for endocrine therapy against breast cancer between 1 January 2020, and 31 December 2020. To increase the likelihood of including only postmenopausal women, and thereby minimize confounding by endogenous estrogen fluctuations, which independently modulate immune function and COVID-19 susceptibility, participants were required to be aged ≥ 55 years at study inclusion. This threshold was chosen based on the median age of natural menopause in Swedish women (approximately 51–52 years), with a margin to account for late menopause. This approach also aligns with the clinical context, as aromatase inhibitors are indicated only in postmenopausal women. All study subjects were followed up for one year.

The study population included women receiving endocrine therapy during 2020, regardless of the year of breast cancer diagnosis, comprising both patients diagnosed in previous years who remained on long-term adjuvant endocrine therapy and those diagnosed in 2020.

All exposed individuals were further classified according to their cancer stage. Patients with documented distant metastases (M1) were few, and a substantial proportion had missing M-status. Separate analyses were conducted for all groups; however, the main focus was on patients with non-metastatic breast cancer (stage I–III).

**Fig. 1:**
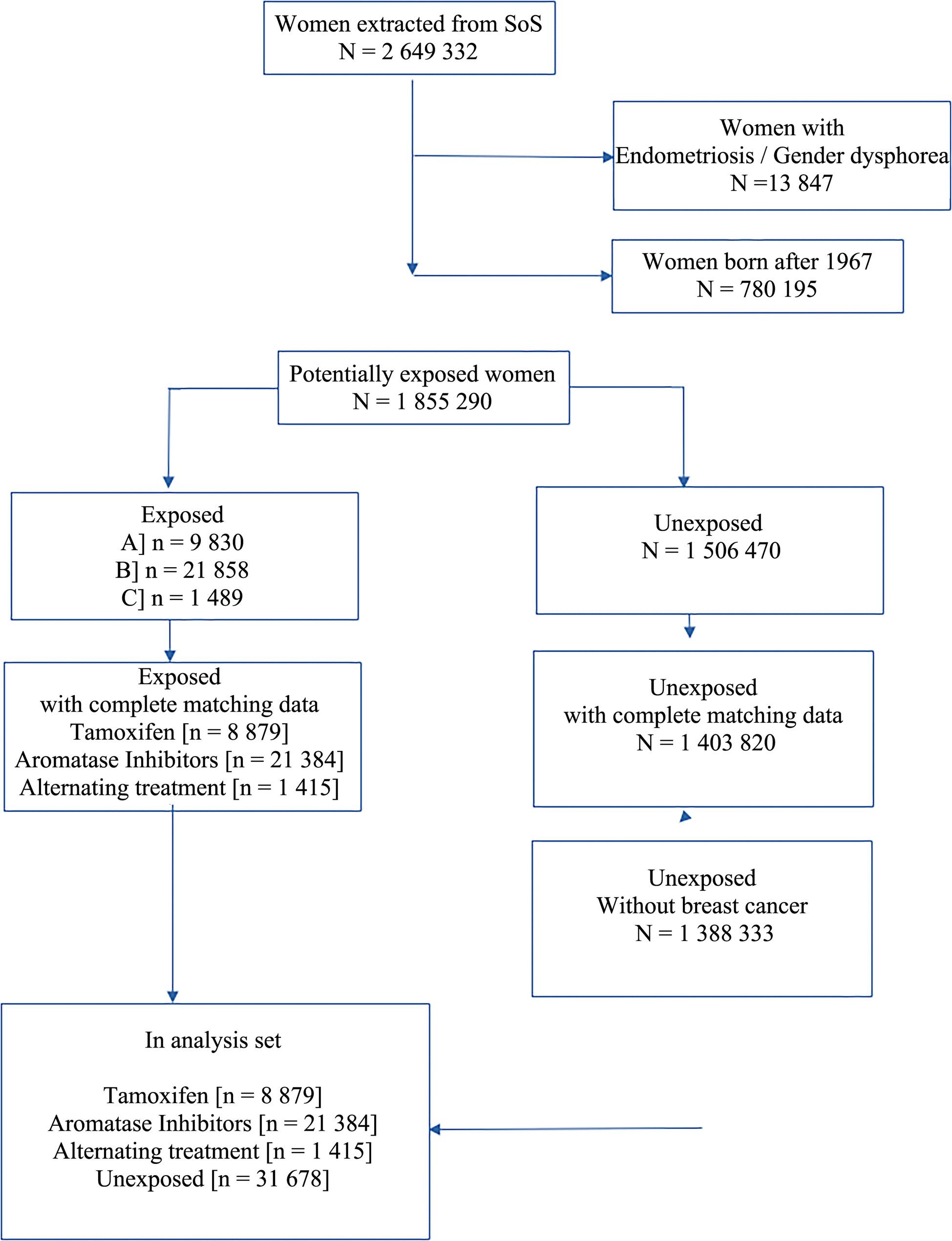
Flowchart of the included population

The unexposed population was randomly selected from the general population and matched for age and residential area (county/region) to exposed individuals at a 1:1 ratio. Non-exposure was defined as being breast cancer–free, without any filled prescriptions for endocrine therapy or any other relevant estrogen-modulating medication (Anatomical Therapeutic Chemical codes: L02AE, G03, G02BA03). Individuals diagnosed with endometriosis [(International Classification of Diseases Version 10 (ICD-10-SE) N80] or gender dysphoria (ICD-10-SE F640, F648, F649) up to five years before the study period were excluded. A flowchart illustrating the included population is shown in Fig. 1.

### Exposure

Exposure status was based on treatment data retrieved from the Prescribed Drug Register. Exposure was defined as having at least one filled prescription during 2020, based on the following Anatomical Therapeutic Chemical codes (ATC):

1. Tamoxifen group: Women receiving tamoxifen (ATC L02BA01).
2. Aromatase inhibitor group: Women receiving aromatase inhibitors (ATC L02BG).
3. Sequential therapy group: Women who alternated between tamoxifen and aromatase inhibitors during follow-up (ATC L02BA01, L02BG).

### Outcomes

Outcomes were registered during 2020 and classified in order of severity as follows:

1. COVID-19–related mortality (primary outcome): Death due to COVID-19 as the main or underlying cause, recorded in the Cause of Death Register (ICD-10-SE U07.1, U07.2).
2. All-cause mortality: Death from any cause recorded in the Cause of Death Register.
3. Intensive care unit admission: Admission due to COVID-19, recorded in the Swedish Intensive Care Register.
4. COVID-19–related hospitalization: Outpatient visits or inpatient hospitalization due to COVID-19 (ICD-10 codes U07.1, U07.2), with or without intensive care unit admission, recorded in the National Patient Register.
5. Laboratory-confirmed SARS-CoV-2 infection: Recorded in Sminet.

Outcomes were evaluated separately, meaning that individuals could contribute to multiple outcome categories. For example, a person who was hospitalized and subsequently died due to COVID-19, was included in both the COVID-19–related mortality category and the hospitalization category. Similarly, all documented COVID-19–related hospitalizations and outpatient visits were also counted as laboratory-confirmed SARS-CoV-2 infections to ensure internal validity between data sources and limit potential misclassification.Follow-up continued until the occurrence of a primary or secondary outcome of interest, death, emigration, or until December 31, 2020, whichever occurred first. Follow-up was concluded before the introduction of COVID-19 vaccines to minimize the potential influence of vaccination on study outcomes.

### Covariates

The following covariates were studied:

- Sociodemographic factors: Age (55–64, 65–74, 75–84, ≥ 85 years), marital status (living with someone versus living alone), education (≤ 12, 13–15, > 15 years), and socioeconomic status (high, middle, working class).
- Comorbidities: Presence of other cancers (yes/no) and comorbidity burden measured using the Uppsala Comorbidity Index, calculated from ICD-10-SE codes registered between 2016 and 2021 in the National Patient Register. The Uppsala Comorbidity Index was categorized as 0, 1, or ≥ 2 with higher values indicating a greater comorbidity burden.

### Statistical analysis

Because the entire eligible populationin Sweden was included, providing optimal power to detect differences in outcomes, no formal power calculation was performed. A complete-case approach was applied in all analyses and no statistical imputation of missing data was performed. To reduce measured confounding and selection bias, propensity score matching was applied to match cancer patients with appropriate controls. Propensity scores were estimated using logistic regression, modeling the probability of exposure as a function of age, marital status, education, socioeconomic status, Uppsala Comorbidity Index score, and presence of other cancers. Matching was conducted using a 1:1 nearest-neighbor approach with a caliper width of 0.2. Balance between groups was assessed using standardized mean differences, with values below 0.1 considered indicative of good balance.

Sociodemographic and background data were presented as rates or median with interquartile range. To explore the association between endocrine therapy and COVID-19 outcomes, logistic regression was used to estimate odds ratios with 95% confidence intervals. Adjusted odds ratios were calculated using logistic regression models that included age, marital status, education, socioeconomic status, Uppsala Comorbidity Index score, and presence of other cancers as covariates.

To assess whether cancer stage modified the association between endocrine therapy and outcomes among women with non-metastatic breast cancer, pre-specified subgroup analyses were conducted according to clinical stage. Patients with distant metastases (M1) were classified as having metastatic disease, and patients with missing or incomplete M-status were categorized as having unknown metastatic status. Analyses were conducted for four groups: early-stage breast cancer (stage I–II), locally advanced breast cancer (stage III), metastatic disease (M1), and unknown metastatic status. Because M1 cases were few and staging information was incomplete for many patients, stage-based analyses focused on women with confirmed non-metastatic disease (stage I–III).

All statistical analyses were performed using R (version 4.3.1). Propensity score matching was carried out with the MatchIt package, and logistic regression was conducted using the glm() function. Statistical significance was set at p 0.05.

## Results

### Demographic characteristics of the study population

The study included 31,678 women treated with endocrine therapy for breast cancer in 2020: 8,879 received tamoxifen, 21,384 received aromatase inhibitors, and 1,415 received sequential therapy. The final matched cohort comprised 63,356 women (exposed and unexposed combined).

Aromatase inhibitor and sequential therapy use increased with age, whereas tamoxifen was more common in younger women. Education levels were similar across treatment groups, with most participants having 13–15 years of education. Among individuals with complete socioeconomic data, socioeconomic status was generally comparable across groups, with the tamoxifen group showing slightly higher proportions of both high- and working-class individuals. Civil status was evenly distributed, with approximately half of the women in each group having a partner. Comorbidity burden was generally low: the majority (76–81%) had an Uppsala Comorbidity Index score of 0. Scores of 1 were observed in 15–18% of the cohort, and only 3–6% had a score of ≥ 2. Other reproductive cancers were rare and evenly distributed across groups. Full background characteristics are presented in Table 1.

**Table 1:** Demographic and clinical characteristics of the cohort

Category	Tamoxifen n(%)	Aromatase inhibitorsn(%)	Sequential therapy^1^ n (%)	Non-exposure^2^ n (%)
All: *n*=63,356				
n	8,879	21, 384	1,415	31,678
*Age (years)*				
55–64	2,848 (32.1 %)	4,673 (21.9 %)	382 (27.0 %)	7,903 (24.9 %)
65-74	3,169 (35.7 %)	7,865 (36.8 %)	546 (38.6 %)	11,580 (36.6 %)
75-84	2,098 (23.6 %)	5,904 (27.6 %)	352 (24.9 %)	8,354 (26.4 %)
≥85	764 (8.6 %)	2,942 (13.7 %)	135 (9.5 %)	3,841 (12.1 %)
*Education*				
Compulsory	1,787 (20.1 %)	5,064 (23.7 %)	302 (21.3 %)	7,885 (24.9 %)
Upper secondary	3,698 (41.6 %)	8,982 (42.0 %)	613 (43.3 %)	13,411 (42.3 %)
Tertiary	3,393 (38.2 %)	7,330 (34.2 %)	499 (35.2 %)	9,877 (31.2 %)
Missing	1 (0.1 %)	8 (0.1 %)	1 (0.2 %)	505 (1.6 %)
*Relationship status*				
Living with someone	4,502 (50.7%)	10,169 (47.5 %)	692 (48.9 %)	14,497 (45.8 %)
Living alone	4,376 (49.2 %)	11,207 (52.4 %)	722 (51.0 %)	16,676 (52.6 %)
*Socioeconomic status*				
Working class	1,555 (17.5 %)	3,131 (14.6 %)	234 (16.6 %)	5,526 (17.4 %)
Middle	613 (6.9 %)	1,219 (5.7 %)	104 (7.3 %)	1,785 (5.6 %)
High	1,585 (17.9 %)	2,728 (12.8 %)	193 (13.6 %)	3,883 (12.3 %)
Missing	5,126 (57.7 %)	14,306 (6.9 %)	884 (62.5 %)	20,484 (64.7 %)
*Obesity*				
Yes	235 (2.6 %)	727 (3.4 %)	46 (3.3 %)	517 (1.6 %)
No	8,644 (97.4 %)	20,657 (96.6 %)	1,369 (96.7 %)	31,161 (98.4 %)
*Alcohol use*				
Yes	45 (0.5 %)	128 (0.6 %)	3 (0.2%)	168 (0.5 %)
No	8,834 (99.5 %)	21,256 (99.4 %)	1,412 (99.8 %)	31,510 (99.5 %)
*Any malignant diagnosis*				
Yes	6,308 (71.0 %)	18,020 (84.3 %)	1,259 (89.0 %)	315 (1.9 %)
No	2,571 (29.0 %)	3,364 (15.7 %)	156 (11.0 %)	31,363 (99.0 %)
*Breast cancer diagnosis*				
Yes	6,204 (69.9 %)	17,807 (83.3 %)	1,255 (88.7 %)	0 (0.0%)
No	2,675 (30.1 %)	3,577 (16.7 %)	160 (10.3%)	31,678 (100.0 %)
*Uppsala Comorbidity Index* ^3^				
0	7,225 (81.4 %)	16,260 (76.0 %)	1,141 (80.6 %)	26,097 (82.4 %)
1	1,299 (14.6 %)	3,918 (18.3 %)	232 (16.4 %)	4,164 (13.1 %)
2+	355 (4.0 %)	1,206 (5.7 %)	42 (3.0 %)	1,417 (4.5 %)

^1^Sequential therapy refers to alternating treatment with both aromatase inhibitors and tamoxifen during follow up

^2^Non-exposure refers to being cancer-free without receiving any hormonal treatment or other estrogen modulating medication

^3^Uppsala Comorbidity Index (UCI) definition in Appendix

### Outcomes

#### Total population

COVID-19-related mortality did not differ significantly between any of the treatment groups and the matched reference population (Table 2). However, all-cause mortality risk was significantly higher in the aromatase inhibitor group (OR = 1.44, 95% CI: 1.31–1.58; adjusted OR = 1.28, 95% CI: 1.16–1.41), but not in the tamoxifen or sequential therapy groups (Table 2).

**Table 2:** COVID-19 related outcomes in the total study population according to type of endocrine therapy. Incidence rates are expressed by 100,000 inhabitants. Relative risk and overall risk are reported with 95% confidence intervals. Adjusted risk estimates were obtained by multivariate regression model controlling for age, marital status, education, socioeconomic status, Uppsala Comorbidity Index score and presence of other cancers

	Tamoxifen	Aromatase inhibitors	Sequential therapy^1^	None-exposure^2^
Total population: 63,356	8,879	21,384	1,415	31,678
*Mortality due to SARS-CoV-2*				
n (deaths)	23	65	3	79
/100.000 inhabitants	259	327	212	NC
RR (95% CIs)	1.04 (0.65-1.65)	1.22 (0.88-1.69)	0.85 (0.27-2.69)	NC
OR (95% CIs)	1.04 (0.64-1.63)	1.22 (0.88-1.69)	0.85 (0.21-2.27)	NC
Adjusted OR (95% CIs)	1.15 (0.70-1.80)	1.05 (0.75-1.46)	0.88 (0.22-2.38)	NC
*All-cause mortality*				
n (deaths)	259	876	40	914
/100.000 inhabitants	2,917	4,097	2,827	NC
RR (95% CIs)	1.01 (0.88-1.16)	**1.42 (1.30-1.56)**	0.98 (0.72-1.34)	NC
OR (95% CIs)	1.01 (0.88-1.16)	**1.44 (1.31-1.58)**	0.98 (0.70-1.33)	NC
Adjusted OR (95% CIs)	1.12 (0.96-1.29)	**1.28 (1.16-1.41)**	1.03 (0.73-1.42)	NC
*ICU admission cause of SARS-CoV-2*				
n	27	76	4	91
/100.000 inhabitants	304	355	283	NC
RR (95% CIs)	1.06 (0.69-1.63)	1.24 (0.91-1.68)	0.98 (0.36-2.67)	NC
OR (95% CIs)	1.06 (0.68-1.60)	1.24 (0.91-1.68)	0.98 (0.30-2.36)	NC
Adjusted OR (95% CIs)	1.13 (0.72-1.72)	1.04 (0.76-1.41)	0.97 (0.30-2.34)	NC
*Outpatient visits or inpatient hospitalization due to SARS-CoV-2*				
n	99	314	18	331
/100.000 inhabitants	1,115	1,468	1,272	NC
RR (95% CIs)	1.07 (0.85-1.33)	**1.41 (1.21-1.64)**	1.22 (0.76-1.95)	NC
OR (95% CIs)	1.07 (0.85-1.33)	**1.41 (1.21-1.65)**	1.22 (0.73-1.91)	NC
Adjusted OR (95% CIs)	1.09 (0.86-1.36)	**1.21 (1.04-1.42)**	1.21 (0.72-1.91)	NC
*Laboratory confirmed SARS-CoV-2 infection*				
n	364	820	55	1,067
/100.000 inhabitants	4,100	3,835	3,887	NC
RR (95% CIs)	**1.22 (1.08-1.37)**	**1.14 (1.04-1.24)**	1.15 (0.88-1.51)	NC
OR (95% CIs)	**1.23 (1.08-1.38)**	**1.14 (1.04-1.25)**	1.16 (0.87-1.51)	NC
Adjusted OR2 (95% CIs)	**1.17 (1.04-1.32)**	1.08 (0.98-1.18)	1.13 (0.85-1.48)	NC

*ICU* Intensive care unit, *AR* Adjusted risk, *CI* Confidence interval, *NC* Not calculated, *OR* Overall risk, *RR* Relative risk

Bold values indicated statistical significance (*p*<0.05)

^1^Sequential therapy refers to alternating treatment with both aromatase inhibitors and tamoxifen during follow up

^2^Non-exposure refers to being cancer-free without receiving any hormonal treatment or other estrogen modulating medication

Intensive care unit admissions were comparable across all groups. COVID-19-related hospitalizations and/or outpatient visits were more prevalent in the aromatase inhibitor group (OR = 1.41, 95% CI: 1.21–1.65; adjusted OR = 1.21, 95% CI: 1.04–1.42). For laboratory-confirmed SARS-CoV-2 infection, patients treated with tamoxifen showed a modest but statistically significant increase in risk (OR = 1.23, 95% CI: 1.08–1.38; adjusted OR = 1.17, 95% CI: 1.04–1.32). The aromatase inhibitor group demonstrated a similar trend that did not however reach statistical significance after adjustment (adjusted OR = 1.08, 95% CI: 0.98–1.18). No significant differences in the risk of laboratory-confirmed infection were observed in the sequential therapy group.

#### Stage-based subgroup analyses

Among the 31,678 exposed women, 252 (0.8%) were classified as having metastatic breast cancer (M1): 23 treated with tamoxifen, 217 with aromatase inhibitors, and 12 with sequential therapy. Stage information was missing for 15,318 women (48.3%). All patients, including those with M1 disease and those with missing staging information, were included in the dataset and analyzed. However, the primary stage-based analyses focused on women receiving adjuvant endocrine therapy for non-metastatic breast cancer (stage I–III) (Tables 2, 3 and 4), results for metastatic and unknown-stage patients are presented in Supplementary Tables 1 and 2. The remaining non-metastatic cohort consisted of 16,108 women with confirmed M0 disease, of whom 98% were classified as having early-stage breast cancer (Stage I–II) and the remainder as having locally advanced breast cancer (Stage III). 

**Table 3:** Outcome among patients treated with endocrine therapy due to early breast cancer. Incidence rates are expressed by 100,000 inhabitants. Relative risk and overall risk are reported with 95% confidence intervals. Adjusted risk estimates were obtained by multivariate regression model controlling for age, marital status, education, socioeconomic status, Uppsala Comorbidity Index score and presence of other cancers

	Tamoxifen	Aromatase inhibitors	Sequential therapy^1^
Total population: 15,635	4,117	10,689	829
*Mortality due to SARS-CoV-2*			
n (deaths)	10	28	2
/100.000 inhabitants	243	262	241
RR (95% CIs)	0.97 (0. 50-1.88)	1.05 (0.68-1.62)	0.97 (0.24-3.93)
OR (95% CIs)	0.97 (0. 47-1.79)	1.05 (0.67-1.60)	0.97 (0.16-3.07)
Adjusted OR (95% CIs)	1.06 (0.51-1.95)	0.95 (0.60-1.45)	1.07 (0.17-3.43)
*All-cause mortality*			
n (deaths)	85	337	15
/100.000 inhabitants	2,065	3,153	1,809
RR (95% CIs)	**0.72 (0.57-0.89)**	1.09 (0.97-1.24)	0.63 (0.38-1.04)
OR (95% CIs)	**0.71 (0.56-0.88)**	1.10 (0.96-1.24)	0.62 (0.35-1.00)
Adjusted OR (95% CIs)	**0.76 (0.60-0.95)**	1.00 (0.88-1.14)	0.69 (0.39-1.12)
*ICU admission cause of SARS-CoV-2*			
n	12	36	3
/100.000 inhabitants	291	337	362
RR (95% CIs)	1.01 (0.56-1.85)	1.17 (0.80-1.72)	1.26 (0.40-3.97)
OR (95% CIs)	1.01 (0.53-1.78)	1.17 (0.79-1.71)	1.26 (0.31-3.36)
Adjusted OR (95% CIs)	1.05 (0.54-1.84)	1.02 (0.68-1.49)	1.29 (0.31-3.48)
*Outpatient visits or inpatient hospitalization due to SARS-CoV-2*			
n	49	137	10
/100.000 inhabitants	1,190	1,282	1,206
RR (95% CIs)	1.14 (0.85-1.53)	**1.23 (1.01-1.50)**	1.15 (0.62-2.16)
OR (95% CIs)	1.14 (0.83-1.53)	**1.23 (1.01-1.50)**	1.16 (0.57-2.06)
Adjusted OR (95% CIs)	1.13 (0.82-1.52)	1.08 (0.88-1.32)	1.17 (0.58-2.09)
*Laboratory confirmed SARS-CoV-2 infection*			
n	162	398	34
/100.000 inhabitants	3,935	3,723	4,101
RR (95% CIs)	1.17 (0.99-1.37)	1.11 (0.99-1.24)	1.22 (0.87-1.70)
OR (95% CIs)	1.18 (0.99-1.39)	1.11 (0.99-1.25)	1.23 (0.85-1.71)
Adjusted OR (95% CIs)	1.11 (0.93-1.31)	1.05 (0.93-1.18)	1.19 (0.82-1.65)

*ICU* Intensive care unit, *AR* Adjusted risk, *CI* Confidence interval, *OR* Overall risk, *RR* Relative risk

Bold values indicated statistical significance (*p*<0.05)

^1^Sequential therapy refers to alternating treatment with both aromatase inhibitors and tamoxifen during follow up

**Table 4:** Outcome among patients treated with endocrine therapy due to locally advanced breast cancer. Incidence rates are expressed by 100,000 inhabitants. Relative risk and overall risk are reported with 95% confidence intervals. Adjusted risk estimates were obtained by multivariate regression model controlling for age, marital status, education, socioeconomic status, Uppsala Comorbidity Index score and presence of other cancers

	Tamoxifen	Aromatase inhibitors	Sequential therapy^1^
Total population: 463	49	401	23
*Mortality due to SARS-CoV-2*			
n (deaths)	0	2	0
/100.000 inhabitants	0	499	0
RR (95% CIs)	NC	2.00 (0.49-8.11)	NC
OR (95% CIs)	NC	2.00 (0.33-6.39)	NC
Adjusted OR (95% CIs)	NC	1.29 (0.21-4.17)	NC
*All-cause mortality*			
n (deaths)	5	40	3
/100.000 inhabitants	10,204	9,975	13,043
RR (95% CIs)	**3.54 (1.54-8.14)**	**3.46 (2.56-4.67)**	**4.52 (1.57-13.01)**
OR (95% CIs)	**3.82 (1.32-8.79)**	**3.73 (2.63-5.14)**	**5.05 (1.19-14.76)**
Adjusted OR (95% CIs)	**3.76 (1.24-9.26)**	**2.79 (1.93-3.92)**	4.04 (0.89-12.97)
*ICU admission cause of SARS-CoV-2*			
n	0	2	0
/100.000 inhabitants	0	499	0
RR (95% CIs)	NC	1.74 (0.43-7.02)	NC
OR (95% CIs)	NC	1.74 (0.29-5.52)	NC
Adjusted OR (95% CIs)	NC	1.13 (0.18-3.65)	NC
*Outpatient visits or inpatient hospitalization due to SARS-CoV-2*			
n	1	12	0
/100.000 inhabitants	2,041	2,993	0
RR (95% CIs)	1.95 (0.28-13.63)	**2.86 (1.62-5.05)**	NC
OR (95% CIs)	1.97 (0.11-9.04)	**2.92 (1.54-5.01)**	NC
Adjusted OR (95% CIs)	1.77 (0.10-8.37)	**2.16 (1.13-3.74)**	NC
*Laboratory confirmed SARS-CoV-2 infection*			
n	1	23	0
/100.000 inhabitants	2,041	5,736	0
RR (95% CIs)	0.61 (0.09-4.22)	**1.70 (1.14-2.54)**	NC
OR (95% CIs)	0.60 (0.03-2.73)	**1.75 (1.11-2.61)**	NC
Adjusted OR (95% CIs)	0.56 (0.03-2.56)	1.55 (0.98-2.32)	NC

*ICU* Intensive care unit, *AR* Adjusted risk, *CI* Confidence interval, *NC* Not calculated, *OR* Overall risk, *RR* Relative risk

Bold values indicated statistical significance (*p*<0.05)

^1^Sequential therapy refers to alternating treatment with both aromatase inhibitors and tamoxifen during follow

#### Early-stage breast cancer

Among women with early-stage breast cancer, the risk of COVID-19–related mortality was low and did not differ across endocrine therapy groups. Similarly, no differences were observed in all-cause mortality, except for tamoxifen, which was associated with a significantly lower risk for death (OR = 0.71, 95% CI: 0.56–0.88; adjusted OR = 0.76, 95% CI: 0.60–0.95). No significant differences were observed in intensive care unit admission, COVID-19–related hospitalization, or laboratory-confirmed SARS-CoV-2 infection across treatment groups (Table 3).

#### Locally advanced breast cancer

Among women with locally advanced breast cancer, COVID-19–related mortality did not differ between treatment groups. However, the risk of all-cause mortality was significantly higher for those receiving tamoxifen (OR = 3.82, 95% CI: 1.32–8.79; adjusted OR = 3.76, 95% CI: 1.24–9.26) and aromatase inhibitors (OR = 3.73, 95% CI: 2.63–5.14; adjusted OR = 2.79, 95% CI: 1.93–3.92). Regarding other COVID-19–related outcomes, women treated with aromatase inhibitors had an increased risk of COVID-19–related hospitalization (OR = 2.92, 95% CI: 1.54–5.01; adjusted OR = 2.16, 95% CI: 1.13–3.74), whereas no significant associations were observed for intensive care unit admission or SARS-CoV-2 infection (Table 4).

#### Metastatic and unknown stage

Despite small group sizes and substantial missing data, results in women with metastatic disease and those with unknown stage were generally consistent with the findings from the overall cohort (Supplementary Tables 1 and 2).

## Discussion

In this nationwide matched cohort study, the association between endocrine therapy for breast cancer and COVID-19 outcomes was evaluated among women with, early-stage, locally advanced, and metastatic disease during the first year of the pandemic. Using Swedish national register data and propensity score matching, no evidence was found that endocrine therapy was associated with an increased risk of COVID-19–specific mortality or intensive care unit admission when compared with matched controls from the general population.

While overall COVID-19 mortality did not differ across therapy groups, stage-specific differences in all-cause mortality and hospitalization patterns were observed: tamoxifen was associated with lower all-cause mortality in early-stage disease, whereas both tamoxifen and aromatase inhibitors were linked to higher all-cause mortality in locally advanced disease, with aromatase inhibitors also associated with increased COVID-19-related hospitalization. The comparable intensive care unit admission risk likely reflects standardized admission criteria during the early pandemic phase.

Although the initial intention was to assess COVID-19–related risks across the entire breast cancer population receiving endocrine therapy in Sweden, the number of patients with documented metastatic disease (M1) was small, and a substantial proportion had missing M-status. Thus, the most robust conclusions apply to non-metastatic breast cancer (stage I–III), whereas results for metastatic or unknown stage should be interpreted cautiously.

During the early phase of the pandemic, international guidelines recommended minimizing immunosuppressive regimens [11], leading many countries to shift toward broader use of neoadjuvant endocrine therapy to safely delay surgery [10]. In contrast, this strategy was only partly implemented in Sweden, where surgical pathways were largely maintainedand adjuvant endocrine therapy was largely continued as standard care. This provided an opportunity to assess its safety in real-world settings. Because the exposure definition was based on endocrine therapy dispensed during 2020, the majority of included women were already receiving adjuvant endocrine therapy prior to the onset of the pandemic. Thus, the study population predominantly reflects women treated in the adjuvant, non-immunosuppressive setting, aligning closely with our aim to evaluate the association between adjuvant endocrine therapy and COVID-19–related outcomes in curative early breast cancer.

### Interpretation of the findings

#### Tamoxifen and lower all-cause mortality in early-stage breast cancer

The observed association between tamoxifen and lower all-cause mortality in early-stage breast cancer compared to the reference population, may be explained by the drug’s immunomodulatory and antiviral properties. Tamoxifen potentiates innate immunity through estrogen receptor–independent pathways, enhancing macrophage phagocytosis and neutrophil function [21–23]. Additionally, tamoxifen demonstrates direct anti-SARS-CoV-2 activity in preclinical models, reducing viral replication and pulmonary inflammation [24, 25]. These findings are consistent with real-world evidence from Italy, where tamoxifen was the only hormonal agent associated with a significant reduction in standardized mortality compared to the regional population [16]. In addition, tamoxifen use has been associated with fewer fatal cardiovascular events (myocardial infarctions) in early randomized controlled trials, a finding subsequently confirmed in meta-analyses compared with placebo or no treatment [26–28]. Potential mechanism includes a reduction in plasma lipid levels (such as total and LDL cholesterol), inhibition of LDL oxidation as well as anti-inflammatory effects, such as decreased C-reactive protein and fibrinogen levels, collectively contributing to a cardioprotective effect against atherogenesis [29–31].

However, due to the observational design, causal inference cannot be established. Alternative explanations include confounding by indication and differences in prescription patterns with tamoxifen preferentially prescribed to younger (premenopausal) or healthier postmenopausal women, with non-advanced tumors. Residual confounding from unmeasured covariates (such as body mass index, frailty, lifestyle factors), as well as a greater adherence to protective behaviors among cancer patients, may also have influenced the results. The modestly increased risk of laboratory-confirmed SARS-CoV-2 infection among tamoxifen users likely reflects surveillance bias rather than true increased susceptibility, as these patients did not exhibit higher COVID-19 mortality, intensive care unit admission or hospitalization, as would be expected if susceptibility were increased.

In contrast, women on tamoxifen with locally advanced cancer exhibited higher all-cause mortality, likely reflecting underlying disease severity rather than being an effect of tamoxifen. This group has a higher baseline mortality risk and treatment decisions may partly reflect frailty or palliative intent, rather than disease alone, which can introduce confounding by indication. Residual confounding from unmeasured factors cannot be excluded, despite adjustment and propensity score matching. Lastly tamoxifen is associated with an increased risk of thromboembolism, which may be particularly relevant in advanced cancer and could have contributed to the observed findings [32].

#### Aromatase inhibitors and higher all-cause mortality/hospitalization in locally advanced disease

The higher all-cause mortality and COVID-19–related hospitalization among women receiving aromatase inhibitors, particularly in locally advanced disease, may reflect both biological and confounding factors. Aromatase inhibitors markedly suppress estrogen, which may promote a pro-inflammatory state, impair immune responses, and remove estrogen’s protective effects against SARS-CoV-2–induced endothelial dysfunction and cytokine activation [33–36]. Consistent with this, menopausal hormone therapy has been associated with lower COVID-19 mortality, supporting a potential protective role of estrogen [37].

However, confounding by indication and disease burden likely drives the observed associations. Aromatase inhibitors are preferentially prescribed to older postmenopausal women with higher comorbidity burden, and locally advanced disease is inherently associated with greater systemic inflammation and frailty. Large-scale evidence supports this interpretation: a JAMA Network Open meta-analysis found that endocrine therapy had the lowest pooled case fatality rate (11%) among all cancer treatments [38], and Chavez-MacGregor et al. reported that endocrine therapy alone was not associated with increased COVID-19 mortality or intensive care unit admission, in contrast to chemotherapy [9]. The prospective NCCAPS study similarly confirmed that chemotherapy, but not endocrine therapy, was associated with higher COVID-19 hospitalization risk [39]. These findings likely reflect a combination of biological effects of estrogen suppression and confounding by disease burden and patient characteristics, with the latter being the predominant explanation.

#### Strengths and limitations

This study has several strengths. The nationwide coverage and complete prescription data, combined with linkage across high-quality registers, minimized selection bias. The matched cohort design and adjustment for multiple covariates (age, marital status, education, socioeconomic status, Uppsala Comorbidity Index score, and presence of other cancers) enhanced internal validity. The study was conducted before the introduction of COVID-19 vaccines, eliminating vaccination as a potential confounder. Additionally, Sweden’s unique pandemic response, maintaining standard cancer care pathways without aggressive lockdowns, provided an opportunity to assess the real-world safety of adjuvant endocrine therapy during active viral circulation.

Several limitations should be acknowledged. First, stage information was missing for nearly half of the exposed cohort (48.3%), and the number of patients with documented metastatic disease was small (*n* = 252), limiting the generalizability of stage-specific findings. Second, potential residual confounding from unmeasured factors, including body mass index, frailty, lifestyle factors, and other immune-modulating treatments, cannot be excluded. Third, surveillance bias and limited early testing capacity during 2020 may have affected SARS-CoV-2 infection risk estimates potentially explaining the modestly increased infection risk observed among tamoxifen users. Fourth, the exposure definition required only one filled prescription during 2020, which may have included non-adherent or temporary users; future studies with access to longitudinal prescription data could consider a sensitivity analysis requiring at least two dispensed prescriptions to better capture continuous use. Finally, results are limited to the pre-vaccination phase and may not generalize to later pandemic periods or to populations with different healthcare systems.

In conclusion, adjuvant endocrine therapy appears safe in non-metastatic breast cancer with respect to COVID-19–specific outcomes. Tamoxifen may confer a protective effect on all-cause mortality in non-metastatic breast cancer, potentially through immunomodulatory and antiviral mechanisms, whereas aromatase inhibitors were associated with higher all-cause mortality and COVID-19–related hospitalization in locally advanced disease, likely reflecting a combination of disease burden and the biological effects of profound estrogen suppression. These findings support the continuation of adjuvant endocrine therapy during pandemic conditions, emphasizing stage-specific risk assessment and careful monitoring for higher-risk groups. As future pandemics remain a possibility, these data provide reassurance that standard adjuvant endocrine therapy for breast cancer can be safely maintained without increased COVID-19 mortality risk.

## Supplementary Information

Supplementary Material 1.

Supplementary Material 2.

## Data Availability

Access to some or all data generated or analyzed in this study is restricted to protect patient confidentiality or due to licensing agreements. Upon request, the corresponding author can provide details on these restrictions and the conditions under which certain data may be made available.

## Appendix

Uppsala Comorbidity Index [UCI] components:

- Congestive heart failure [I43, I50, I099, I110, I130, I132, I255, I420, I425-I429],
- Dementia [F00-F03, G30, G311, F051],
- Mild liver disease [B18, K73, K74, K700-K703, K709, K713-K715, K717, K760, K762 - K764, K768, K769, Z944],
- Moderate to severe liver disease [K704, K711, K721, K729, K765 - K767, I850, I859, I864, I892], Paraplegia & hemiplegia [G81, G82, G041, G114, G801, G802, G830 – G834, G839],
- Cancer other than breast cancer [C.*, except C50],
- Aids & HIV [B20-B22, B24].

Comorbidities [ICD-10 codes from National Patient Register, 2016–2021]:

Alcohol abuse [F10.2], Obesity [E66], Uppsala Comorbidity Index [UCI] shown above.

## Abbreviations

CI: Confidence interval
COVID-19: Coronavirus disease 2019
ICD: International Classification of Diseases
OR: Odds ratio
SARS-CoV-2: Severe acute respiratory syndrome coronavirus 2
SMD: Standardized mean difference
UCI: Uppsala Comorbidity Index
