# Supplementary material for: Endocrine therapy and COVID-19 outcomes in women with breast cancer: a nationwide register- based matched cohort study
Source: BMC Cancer. 2026 May 12;26:598. doi: 10.1186/s12885-026-16152-6 (PMC13162516; doi:10.1186/s12885-026-16152-6)
Supplement: Supplementary file 1 — Supplementary Material 1. [file 12885_2026_16152_MOESM1_ESM.docx]

# STROBE Checklist – Endocrine Therapy and COVID-19 Outcomes

**STROBE Statement — Checklist of items that should be included in reports of cohort studies**

| **Item No.** | **Section** | **Recommendation** | **Reported on Page** |
| --- | --- | --- | --- |
| Title and Abstract |  |  |  |
| 1 | Title and abstract | (a) Indicate the study's design with a commonly used term in the title or the abstract; (b) Provide in the abstract an informative and balanced summary of what was done and what was found | Title: "Nationwide Register-Based Matched Cohort Study"; Abstract (page 1): Design, population, exposures, outcomes, and key findings summarized |
| Introduction |  |  |  |
| 2 | Background/rationale | Explain the scientific background and rationale for the investigation being reported | Introduction, pages 3–4: Pandemic disruption of cancer care, biological rationale for ET–immune interactions, conflicting prior evidence |
| 3 | Objectives | State specific objectives, including any prespecified hypotheses | Introduction, page 4: Evaluate association between tamoxifen or aromatase inhibitors and COVID-19 outcomes with focus on stage-specific differences; no prespecified hypothesis formulated |
| Methods |  |  |  |
| 4 | Study design | Present key elements of study design early in the paper | Methods: Study design and data sources, page 5: "nationwide, observational, register-based matched cohort study" |
| 5 | Setting | Describe the setting, locations, and relevant dates, including periods of recruitment, exposure, follow-up, and data collection | Methods: Study design and data sources; Study population, pages 5–6: Sweden nationwide; inclusion 1 January–31 December 2020; one-year follow-up; concluded before COVID-19 vaccine introduction |
| 6 | Participants | (a) Give the eligibility criteria, and the sources and methods of selection of participants. Describe methods of follow-up; (b) For matched studies, give matching criteria and number of exposed and unexposed | Methods: Study population, pages 6–7: Women aged ≥55 with ≥1 filled ET prescription; matched 1:1 by age and region; 31,678 exposed and 31,678 unexposed; exclusions described; Figure 1 flowchart |
| 7 | Variables | Clearly define all outcomes, exposures, predictors, potential confounders, and effect modifiers. Give diagnostic criteria, if applicable | Methods: Exposure; Outcomes; Covariates, pages 7–9: ATC codes for exposure groups; five outcomes with ICD-10 codes and data sources; covariates with categories defined |
| 8 | Data sources/measurement | For each variable of interest, give sources of data and details of methods of assessment (measurement). Describe comparability of assessment methods if there is more than one group | Methods: Study design and data sources; Outcomes, pages 5–6, 8: Eight national registers described with providing agencies; each outcome linked to specific register |
| 9 | Bias | Describe any efforts to address potential sources of bias | Methods: Statistical analysis, page 9: Propensity score matching (1:1, caliper 0.2), adjusted logistic regression; Discussion — Strengths and limitations, pages 14–15: Residual confounding, surveillance bias acknowledged |
| 10 | Study size | Explain how the study size was arrived at | Methods: Statistical analysis, page 9: Entire eligible population included; no formal power calculation performed |
| 11 | Quantitative variables | Explain how quantitative variables were handled in the analyses. If applicable, describe which groupings were chosen and why | Methods: Covariates, page 8: Age (55–64, 65–74, 75–84, ≥85), education (≤12, 13–15, >15 years), UCI (0, 1, ≥2) |
| 12 | Statistical methods | (a) Describe all statistical methods, including those used to control for confounding; (b) Describe any methods used to examine subgroups and interactions; (c) Explain how missing data were addressed; (d) If applicable, describe analytical methods taking account of sampling strategy; (e) Describe any sensitivity analyses | Methods: Statistical analysis, pages 9–10: (a) Propensity score matching and adjusted logistic regression with specified covariates; (b) Pre-specified subgroup analyses by stage; (c) Complete-case approach, no imputation; (d) Not applicable; (e) Sensitivity analysis with ≥2 prescriptions acknowledged as future direction in Limitations |
| Results |  |  |  |
| 13 | Participants | (a) Report numbers of individuals at each stage of study — e.g., numbers potentially eligible, examined for eligibility, confirmed eligible, included in the study, completing follow-up, and analysed; (b) Give reasons for non-participation at each stage; (c) Consider use of a flow diagram | Results: Demographic characteristics, pages 10–11: 31,678 exposed (8,879 tamoxifen, 21,384 AI, 1,415 sequential); 63,356 total. Stage subgroups: 16,108 M0, 252 M1, 15,318 missing stage. Figure 1 flowchart |
| 14 | Descriptive data | (a) Give characteristics of study participants (e.g., demographic, clinical, social) and information on exposures and potential confounders; (b) Indicate number of participants with missing data for each variable of interest; (c) Summarise follow-up time (e.g., average and total amount) | Results: Demographic characteristics; Table 1, pages 10–11: Age, education, relationship status, socioeconomic status, obesity, alcohol use, cancer diagnoses, UCI by group. Missing data reported for education, socioeconomic status (Table 1), and stage (48.3%, Results). Follow-up: 1 January–31 December 2020 |
| 15 | Outcome data | Report numbers of outcome events or summary measures over time | Results: Outcomes; Tables 2–4, pages 11–13: Number of events, incidence per 100,000, RR, OR, and adjusted OR with 95% CIs for all five outcomes across treatment groups |
| 16 | Main results | (a) Give unadjusted estimates and, if applicable, confounder-adjusted estimates and their precision (e.g., 95% confidence interval). Make clear which confounders were adjusted for and why they were included; (b) Report category boundaries when continuous variables were categorized; (c) If relevant, consider translating estimates of relative risk into absolute risk for a meaningful time period | Results: Outcomes; Tables 2–4, pages 11–13: Unadjusted and adjusted ORs with 95% CIs. Adjustment variables specified in table footnotes and Methods. Incidence per 100,000 reported alongside relative measures |
| 17 | Other analyses | Report other analyses done — e.g., analyses of subgroups and interactions, and sensitivity analyses | Results: Stage-based subgroup analyses; Tables 3–4; Supplementary Tables 1–2, pages 12–13: EsBC, LABC, metastatic, and unknown stage analysed separately |
| Discussion |  |  |  |
| 18 | Key results | Summarise key results with reference to study objectives | Discussion, page 14: No increased COVID-19 mortality or ICU admission with ET; stage-specific differences in all-cause mortality and hospitalization |
| 19 | Limitations | Discuss limitations of the study, taking into account sources of potential bias or imprecision. Discuss both direction and magnitude of any potential bias | Discussion: Strengths and limitations, pages 14–15: Missing stage data (48.3%), small M1 group, residual confounding, surveillance bias, single-prescription exposure, pre-vaccination period |
| 20 | Interpretation | Give a cautious overall interpretation of results considering objectives, limitations, multiplicity of analyses, results from similar studies, and other relevant evidence | Discussion: Interpretation of the findings, pages 14–15: Biological rationale for tamoxifen and AI findings; comparison with Bravaccini et al., Chavez-MacGregor et al., NCCAPS, Khoury et al.; causal limitations stated |
| 21 | Generalisability | Discuss the generalisability (external validity) of the study results | Discussion: Strengths and limitations, page 15: Results limited to pre-vaccination phase; may not generalize to other pandemic periods or healthcare systems |
| Other Information |  |  |  |
| 22 | Funding | Give the source of funding and the role of the funders for the present study and, if applicable, for the original study on which the present article is based | Funding section, page 16: Uppsala University Hospital (ALF grants) and Oncology Foundation in Uppsala; funders had no role in study design, data collection, analysis, or manuscript preparation |
