# Supplementary material for: Endocrine therapy and COVID-19 outcomes in women with breast cancer: a nationwide register- based matched cohort study
Source: BMC Cancer. 2026 May 12;26:598. doi: 10.1186/s12885-026-16152-6 (PMC13162516; doi:10.1186/s12885-026-16152-6)
Supplement: Supplementary file 2 — Supplementary Material 2. [file 12885_2026_16152_MOESM2_ESM.docx]

|  |  | Tamoxifen | Aromatase inhibitors | Sequential therapy^1^ |
| --- | --- | --- | --- | --- |
| Total population: 252 | | 23 | 217 | 12 |
| *Mortality due to SARS-CoV-2* | |  |  |  |
|  | n (deaths) | 0 | 2 | 1 |
|  | /100.000 inhabitants | 0 | 922 | 8,333 |
|  | RR (95% CIs) | NC | 3.70 (0.91-14. 94) | **33.42 (5.05-221.07)** |
|  | OR (95% CIs) | NC | 3.72 (0.61-11. 90) | **36.36 (1.98-190.28)** |
|  | Adjusted OR (95% CIs) | NC | 2.32 (0.38-7.57) | **22.17 (1.18-122.49)** |
| *All-cause mortality* | |  |  |  |
|  | n (deaths) | 9 | 33 | 2 |
|  | /100.000 inhabitants | 39,130 | 15,207 | 16,667 |
|  | RR (95% CIs) | **13.56 (8.11-22.67)** | **5.27 (3.82-7.26)** | **5.78 (1.63-20.50)** |
|  | OR (95% CIs) | **21.64 (8.99-49.46)** | **6.04 (4.07-8.67)** | **6.73 (1.03-25.58)** |
|  | Adjusted OR (95% CIs) | **17.66 (6.80-43.92)** | **4.68 (3.06-6.96)** | 4.63 (0.69-18.34) |
| *ICU admission cause of SARS-CoV-2* | | |  |  |
|  | n | 0 | 2 | 1 |
|  | /100.000 inhabitants | 0 | 922 | 8,333 |
|  | RR (95% CIs) | NC | 3.21 (0.80-12. 94) | **29.01 (4.39-191.59)** |
|  | OR (95% CIs) | NC | 3.23 (0.53-10. 29) | **31.56 (1.72-164.73)** |
|  | Adjusted OR (95% CIs) | NC | 2.09 (0.34-6.79) | 0.97 (0.30-2.34) |
| *Outpatient visits or inpatient hospitalization due to SARS-CoV-2* | | |  |  |
|  | n | 0 | 6 | 1 |
|  | /100.000 inhabitants | 0 | 2,765 | 8,333 |
|  | RR (95% CIs) | NC | **2.65 (1.19-5.87)** | **7.98 (1.22-52.25)** |
|  | OR (95% CIs) | NC | **2.69 (1.06-5.59)** | 8.61 (0.47-44.44) |
|  | Adjusted OR (95% CIs) | NC | 1.93 (0.75-4.05) | 8.27 (0.45-44.22) |
| *Laboratory confirmed SARS-CoV-2 infection* | | |  |  |
|  | n | 1 | 8 | 2 |
|  | /100.000 inhabitants | 4,348 | 3,687 | 16,667 |
|  | RR (95% CIs) | 1.29 (0.19-8.79) | 1.09 (0.55-2.17) | **4.95 (1.39-17.56)** |
|  | OR (95% CIs) | 1.30 (0.07-6.21) | 1.10 (0.50-2.08) | 5.74 (0.88-21.80) |
|  | Adjusted OR (95% CIs) | 1.17 (0.06-5.62) | 0.97 (0.44-1.85) | **6.72 (1.03-25.75)** |

^1^Sequential therapy refers to alternating treatment with both aromatase inhibitors and tamoxifen during follow up.

**Suppl table 1.** Outcome among patients treated due to metastatic breast cancer. Incidence rates are expressed by 100,000 inhabitants. Relative risk and overall risk are reported with 95% confidence intervals. Adjusted risk estimates were obtained by multivariate regression model controlling for age, marital status, education, socioeconomic status, Uppsala Comorbidity Index score and presence of other cancers. AR: Adjusted risk, CI: confidence interval, NC: Not calculated, OR: Overall risk, RR: Relative risk. Bold values indicated statistical significance (p<0.05).

ICU: intensive care unit

|  |  | *Tamoxifen* | *AI* | Sequential therapy^1^ |
| --- | --- | --- | --- | --- |
| Total population: 15,588 | | 4,960 | 10,077 | 551 |
| *Mortality due to SARS-CoV-2* | |  |  |  |
|  | n (deaths) | 13 | 33 | 0 |
|  | /100.000 inhabitants | 277 | 327 | 0 |
|  | RR (95% CIs) | 1.11 (0.62-2.00) | 1.31 (0.88-1.97) | NC |
|  | OR (95% CIs) | 1.11 (0.59-1.93) | 1.31 (0.86-1.95) | NC |
|  | Adjusted OR (95% CIs) | 1.26 (0.67-2.19) | 1.10 (0.72-1.64) | NC |
| *All-cause mortality* | |  |  |  |
|  | n (deaths) | 160 | 466 | 20 |
|  | /100.000 inhabitants | 3,412 | 4,624 | 3,630 |
|  | RR (95% CIs) | **1.18 (1.00-1.39)** | **1.60 (1.44-1.79)** | 1.26 (0.81-1.94) |
|  | OR (95% CIs) | **1. 19 (1.00-1.41)** | **1.63 (1.46-1.83)** | 1.27 (0.78-1.94) |
|  | Adjusted OR (95% CIs) | **1.34 (1.12-1.60)** | **1.43 (1.27-1.61)** | 1.28 (0.78-1.98) |
| *ICU admission cause of SARS-CoV-2* | |  |  |  |
|  | n | 15 | 36 | 0 |
|  | /100.000 inhabitants | 320 | 357 | 0 |
|  | RR (95% CIs) | 1.11 (0.65-1.92) | 1.24 (0.85-1.83) | NC |
|  | OR (95% CIs) | 1.11 (0.62-1.86) | 1.24 (0.84-1.81) | NC |
|  | Adjusted OR (95% CIs) | 1.24 (0.69-2.08) | 1.02 (0.69-1.50) | NC |
| *Outpatient visits or inpatient hospitalization due to SARS-CoV-2* | | |  |  |
|  | n | 49 | 159 | 7 |
|  | /100.000 inhabitants | 1,045 | 1,578 | 1,270 |
|  | RR (95% CIs) | 1.00 (0.74-1.35) | **1.51 (1.25-1.82)** | 1.22 (0.58-2.56) |
|  | OR (95% CIs) | 1.00 (0.73-1.34) | **1.52 (1.25-1.83)** | 1.22 (0.52-2.39) |
|  | Adjusted OR (95% CIs) | 1.04 (0.76-1.40) | **1.29 (1.06-1.57)** | 1.20 (0.51-2.38) |
| *Laboratory confirmed SARS-CoV-2 infection* | | |  |  |
|  | n | 200 | 391 | 19 |
|  | /100.000 inhabitants | 4,264 | 3,880 | 3,448 |
|  | RR (95% CIs) | **1.27 (1.09-1.47)** | **1.15 (1.03-1.29)** | 1.02 (0.66-1.60) |
|  | OR (95% CIs) | **1.28 (1.09-1.49)** | **1.16 (1.03-1.30)** | 1.02 (0.62-1.58) |
|  | Adjusted OR (95% CIs) | **1.24 (1.06-1.44)** | 1.08 (0.98-1.18) | 1.01 (0.62-1.56) |

^1^Sequential therapy refers to alternating treatment with both aromatase inhibitors and tamoxifen during follow up.

**Suppl table 2.** Outcome among patients with breast cancer of unknown stage treated with endocrine treatment. Incidence rates are expressed by 100,000 inhabitants. Adjusted risk estimates were obtained by multivariate regression model controlling for age, marital status, education, socioeconomic status, Uppsala Comorbidity Index score and presence of other cancers. AR: Adjusted risk, CI: confidence interval, NC: Not calculated, OR: Overall risk, RR: Relative risk. Bold values indicated statistical significance (p<0.05).
